# Patient information, communication and competence empowerment in oncology: Results and learnings from the PIKKO study

**DOI:** 10.1007/s00520-023-07781-9

**Published:** 2023-05-08

**Authors:** Nico Schneider, Anna Bäcker, Bernhard Strauss, Jutta Hübner, Sabine Rubai, Steffen Wagner, Doris Schwarz-Fedrow, Lutz Hager, Katja Brenk-Franz, Christian Keinki, Florian Brandt, Uwe Altmann

**Affiliations:** 1 Institute of Psychosocial Medicine, Psychotherapy and Psychoonocology (IPMPP), Jena University Hospital, Jena, Germany; 2 Department of Haematology and Medical Oncology, Jena University Hospital, Jena, Germany; 3 Saarland Cancer Society, Saarbrücken, Germany; 4 SRH Fernhochschule – The Mobile University, Riedlingen, Germany; 5 The German Cancer Society, Berlin, Germany; 6 IKK Südwest, Saarbrücken, Germany; 7 Department of Psychology, MSB Medical School Berlin GmbH, Berlin, Germany

**Keywords:** Cancer, Patient navigator, Psycho-social counseling, Knowledge database, Health-related quality of life

## Abstract

**Purpose:**

Many concepts for accompanying and supporting cancer patients exist and have been studied over time. One of them was PIKKO (a German acronym for “Patient information, communication and competence empowerment in oncology”), which combined a patient navigator, socio-legal and psychological counseling (with psychooncologists), courses dealing with various supportive aspects, and a knowledge database with validated and easy-to-understand disease-related information. The aim was to increase the patients' health-related quality of life (HRQoL), self-efficacy as well as health literacy and to reduce psychological complaints such as depression and anxiety.

**Methods:**

To this purpose, an intervention group was given full access to the modules in addition to treatment as usual, while a control group received only treatment as usual. Over twelve months, each group was surveyed up to five times. Measurements were taken using the SF12, PHQ-9, GAD, GSE, and HLS-EU-Q47.

**Results:**

No significant differences were found in scores on the mentioned metrics. However, each module was used many times and rated positively by the patients. Further analyses showed a tendency higher score in health literacy with higher intensity of use of the database and higher score in mental HRQoL with higher intensity of use of counseling.

**Conclusion:**

The study was affected by several limitations. A lack of randomization, difficulties in recruiting the control group, a heterogeneous sample, and the COVID-19 lockdown influenced the results. Nevertheless, the results show that the PIKKO support was appreciated by the patients and the lack of measurable effects was rather due to the mentioned limitations than to the PIKKO intervention.

**Trial registration:**

This study was retrospectively registered in the German Clinical Trial Register under DRKS00016703 (21.02.2019, retrospectively registered). https://www.drks.de/drks_web/navigate.do?navigationId=trial.HTML&TRIAL_ID=DRKS00016703

## Introduction

While various cancer treatments are constantly evolving, survival rates are increasing [1], and outpatient support services are accumulating, there is often still a lack of information and psycho-social support for oncological patients [2–4]. However, better informing patients leads to greater satisfaction with their treatment and improved quality of life [5]. Patients' needs include high quality, evidence-based, and helpful health information [6, 7], to active participation in decision making [8, 9] and socio-legal [10] and psycho-social support [11]. Beyond the attending physicians, psycho-oncological or socio-legal counselors [12, 13], patient navigators [14–16] and e-health services [17] can all provide helpful information and psycho-social support, thus improving the quality of life.

Currently, psychooncology as part of the German healthcare system is guideline-approved [18] and conducted in both outpatient settings by cancer counseling centers [12, 13] and inpatient settings by psychooncologists employed by hospitals. A meta-analysis (55 studies) of individual psychotherapeutic interventions conducted as under the guidelines showed a significant, heterogeneous, small to medium-sized effect of the interventions on quality of life in the long term (> 6 months) [18]. Effects on anxiety and depression were small but significant in the medium term (≤ 6 months) [18]. A median of six individual sessions over seven weeks was provided [18]. However, utilization is always associated with barriers, partly because due to a lack of knowledge about the interventions available [19, 20].

Patient navigators (PN) help guide a patient through the healthcare system, e.g., by supporting the communication process in order to facilitate shared decision making. They were recently described in a publication by the European Observatory on Health Systems and Policies as a "skill-mix innovation where a new role or tasks are introduced" to meet the increased demands and needs of patients [21]. However, effectiveness has only been partially reported and was limited to specific situations [21]. For example, Lee [22] and Fillion [23] found an increased quality of life. Nevertheless, there have been numerous efforts in Germany to introduce “Onco-navigators” (in German: “Onkolotsen”) to help patients navigate the cancer treatment process, involving several different stakeholders [24, 25]. Already in 2010, the Saxon Cancer Society began implementing onco-navigators [26]. Current projects include "OSCAR" [27], "Familien-SCOUT" [28], "ONCOPATH" [29] and "PIKKO" [30]. The Federal Association of Managed Care (in German: Bundesverband Managed Care (BMC)) provides an overview of PN projects in Germany [25].

**Fig. 1:**
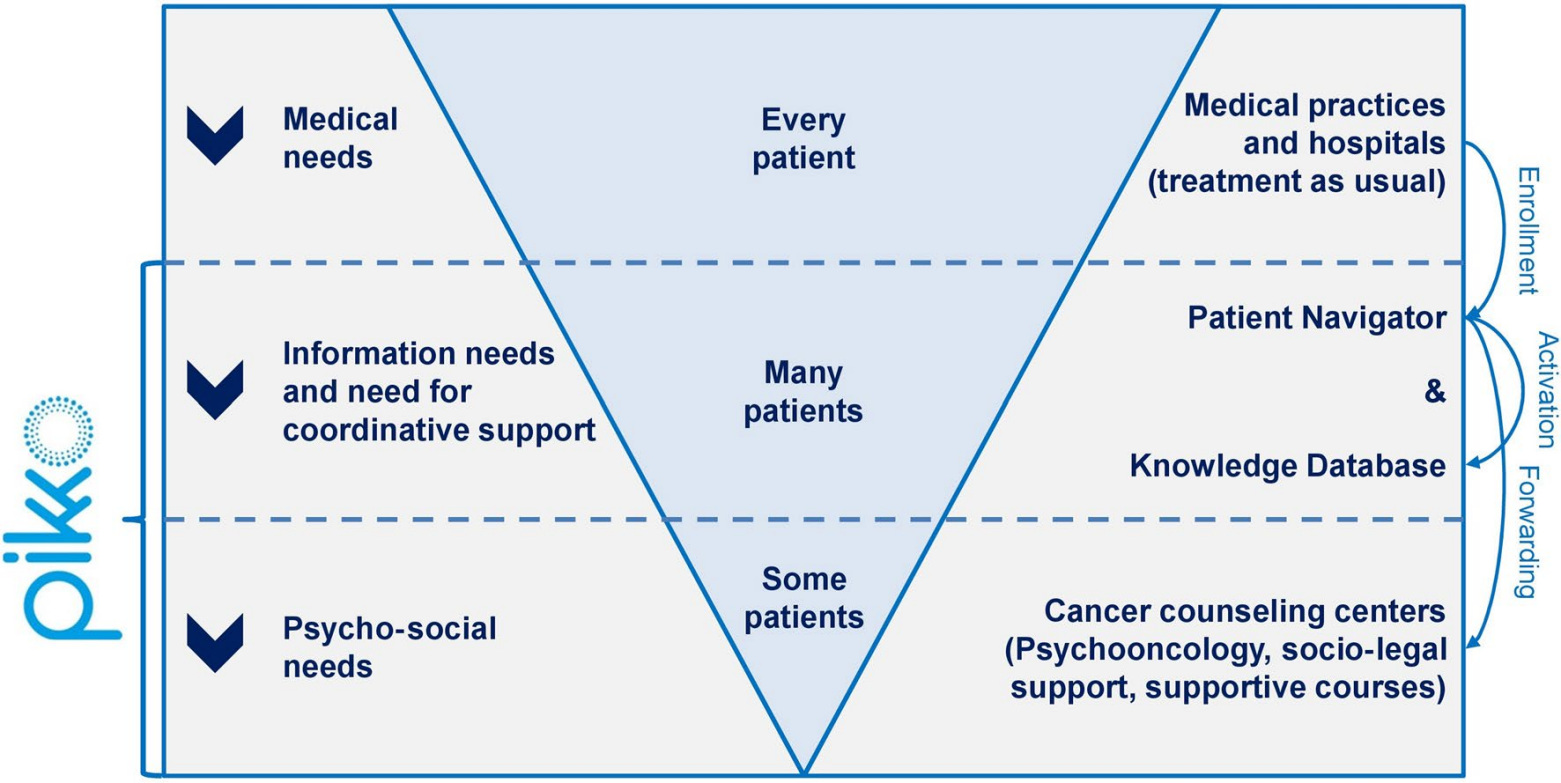
Stepped-care approach and supply process of the PIKKO intervention. Source: Own representation adapted from Fitch, M. (2008): Supportive care framework. Can Oncol Nurs J, 18(1):6–24 × 181,614. https://doi.org/10.5737/1181912

The aim of PIKKO was to combine the effects of a continuously available PN with specialized psycho-oncological and socio-legal counseling and to complement it with a digital information platform that provides oncological and socio-legal content up-to-date and using language understandable for laypeople. All interventions were designed as supplementary to treatment as usual (e.g., chemotherapy or radiotherapy), could be used according to individual needs, and were offered using a stepped-care approach as shown in Fig. 1.

PIKKO was open for the entire spectrum of cancer patients. There was no restriction regarding cancer type or disease stage. The hypotheses was that the use of PIKKO is associated with a higher health-related quality of life, self-efficacy and health literacy as well as lower measures of depression and anxiety.

## Methods

This paper reports the findings of the patient survey, which was the main component of the overall evaluation process within the PIKKO study [30]. The CONSORT Statement for Randomized Trials of Nonpharmacologic Treatments 2017 was used to report this trial [31].

### Trial design

The evaluation of the PIKKO intervention followed a non-randomized, controlled, comparative, multicenter, longitudinal design. Two groups of patients were surveyed one after the other. Group assignment was determined by the date of recruitment: control group (CG) in the first and the beginning of the second project year (while the PIKKO modules were under development) and intervention group (IG) in the second and third project year (with available PIKKO modules).

### Participants

Since this was a health care project initiated by the statutory health insurance companies, only their insured persons could participate. Geographically, the study was limited to Saarland, one of the 16 federal states in Germany with nearly one million inhabitants and more than 8,400 malignant neoplasms in 2019 [32]. Thirty-eight medical institutions of different specialties in 19 cities actively participated in the recruitment. Patients were either undergoing outpatient or inpatient treatment at the time of recruitment. Participants were recruited from physicians working in participating inpatient or outpatient facilities. Physicians were informed about PIKKO by participating statutory health insurance companies and agreed to enroll patients.

The eligibility criteria were:

- age ≥ 18 years and ≤ 90 years,
- diagnosis of any cancer (ICD-10-diagnosis group C00-C97 or D45-D48 (initial diagnosis, relapse or transition to palliative care)),
- no statutory guardianship,
- sufficient knowledge of the German language,
- no severe visual or hearing impairment,
- no dementia or other mental limitations.

All cancer patients that met the inclusion criteria were eligible for participation – no needs screening was performed. The heterogeneity in types of cancer, severity, stages of disease and comorbidities and the plurality of medical institutions should reflect everyday care.

### Intervention

The PIKKO intervention consisted of four modules that every patient of the IG could freely use: the patient navigator (PN), an offer of the Saarland Cancer Society (SCS) consisting of psycho-oncological/social-legal counseling as well as various courses, a web-based knowledge database ("My PIKKO"), and a ring folder. All elements formed an additional information pathway for cancer patients that was not intended to replace usual care.

The PN was a non-physician person with a medical background (medical assistant or nurse) and oncological experience (at least two years) who had received a two-week intensive course from the German Cancer Society (GCS). This course was adapted from the curriculum for PN of the Saxon Cancer Society [33]. The PN primarily had a counseling role, could connect the patient to other counseling services, especially those of the SCS, and gave the patient access to the web-based knowledge database. A total of 15 PN (14 women) were deployed (3 in medical practices and 12 in hospitals).

As described, SCS offered counseling sessions and various supportive courses. Regardless of the status of their disease, patients could contact the SCS psychooncologists and social workers to discuss their psychological or socio-legal problems. Other courses offered to all IG patients included nutrition courses, telephone nutrition counseling, art and creative courses, music therapy, Nordic walking, QiGong, and yoga. All courses were also open to patients outside the PIKKO study, but with a limited quota.

The web-based knowledge database "My PIKKO" was developed, hosted, and continuously updated by the GCS and contained evidence-based general information about cancer, information about patients' specific cancer and socio-legal information. In addition, patients could create checklists for doctor-patient discussions and the database included an address search for available medical services in Saarland. The readability and comprehensibility of the texts were continuously reviewed and adapted to the needs of medical laypersons. After an introduction by the PN, patients had 24-h access to the database.

As an offer to all PIKKO participants (as a small incentive, this was also handed out to the CG), a ring folder was provided, which was specially designed by the GCS for PIKKO and contained important local and regional information (e.g., lists of self-help groups). It was also used for treatment documentation.

All modules could be used freely by patients as needed. There were no obligations and no dose requirements. How often the modules were used was up to the patients themselves, depending on their needs. This reflected the use of daily medical care.

### Outcomes

As stated in the hypotheses, it was hypothesized that participation in the PIKKO intervention is associated with better health-related quality of life six months after inclusion. The main effect was measured six months after baseline using the SF-12 [34], but there were additional measures at three, nine and twelve months. In addition, it was hypothesized that use of the intervention is associated with increased self-efficacy (measured with the GSE [35]), reduced psychological distress such as depression (measured with the PHQ-9 [36]) or generalized anxiety (measured with the GAD-7 [37]), and increased health literacy (measured with the HLS-EU-Q47 [38]).

All questionnaires, together with socio-demographic and disease questions were part of the overall PIKKO questionnaire, which patients completed themselves (see schedule of all survey times in Table 1). A maximum of five (at baseline, after three months, after six months, after nine months and after twelve months) of these surveys were completed per patient within one year. The IG also answered detailed questions about the use of the PIKKO modules. They also rated each intervention module as a whole on a 6-point scale modeled after grading in the German school system, with which each participant was familiar (1 = very good to 6 = insufficient).

**Table 1:**
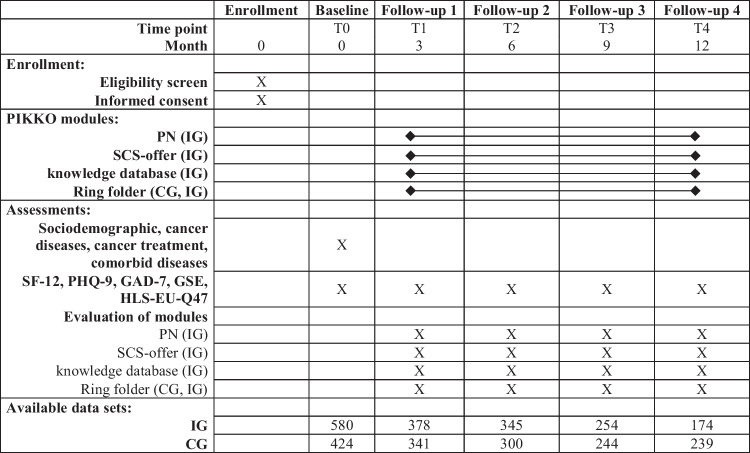
Schedule of enrollment, PIKKO modules, assessments, and available data sets

The use of the knowledge database was analyzed with the help of log files.

### Sample size

The sample size for the evaluation of the PIKKO intervention was set at 1,014 patients. This number was derived from the calculated size of 676 patients (expected effect of d = 0.25 for a two-sided t-test on the primary outcome, with equal sized comparison groups, a significance level of α = 0.05 and a power of 1-β = 0.9; calculated with the program GPower version 3.1.7) plus an additional 338 to compensate for the expected dropout (33.3% with respect to baseline, 338 of 1,014, or 50% with respect to patients who reached the end point, 338 of 676). Because both groups should be of equal size, 507 patients (338 reaching end point + 169 dropouts) per group are targeted.

### Randomization

Random assignment of patients to two parallel groups was not possible for ethical and service reasons, since a health insurance company should not refuse patients to participate in one of its innovative and publicly known health services (such as the PIKKO intervention). For this reason, the PIKKO intervention and the IG survey did not begin until after recruitment of the CG had ended. The assignment of patients to groups depended solely on the time of enrollment.

### Statistical methods

First, for sample characterization and to determine selection effects, univariate analysis of variance and chi-square test were used to compare the groups in essential characteristics (socio-demographic, disease-related parameters).

Before the main analyses, an imputation was performed using the method MissForest (R package) with the following variables (percentage of missing): Age (0.0%), gender (0.0%), marital status (0.2%), children in the household (2.1%), school graduation (0.0%), cancer type (0.0%), cancer age (6.7%), presence of metastases (0.0%), nodus (0.0%), and relapse (0.0%), tumor status (0.0%), comorbid conditions (0.0%), information on treatment at baseline (0.0%), sum scores of questionnaires at baseline and all follow-ups (missings see Table 3), use of intervention modules (missings see Table 4), was the measurement during the COVID-19 lockdown (T1: 28.4%, T2: 35.8%, T3: 50.4%, T4: 58.9%), support for filling in (T0: 7.1%, T1: 31.1%, T2: 36.4%, T3: 51.0%, T4: 59.3%). When applying the missForest algorithm, we set the maximum number of iterations on 30 and the number of trees to grow in each forest on 1,000. The imputation algorithm converged after 19 iterations (normalized root-mean-square error = 0.245, proportion of falsely classified entries = 0.065).

To compensate for the lack of randomization, regression weights were calculated based on stabilized propensity scores [39] using R package WeightIt. The predictors used were: Age, sex, inpatient treatment at enrollment, cancer type (the four most common), cancer age, presence of metastases, nodes, and relapse, tumor status, comorbid diseases, treatment information at baseline and follow-up 1, questionnaire sum scores at baseline.

Regression-adjusted analyses of differences between IG and CG were performed with imputed data. Means and standard deviations were estimated using R package ggeffects and compared using Welch test. Hedges' g is used as the effect size.

Since the use of the intervention modules was voluntary, patients used the modules with varying frequency at the measurement time points. We used growth curve models [40] to examine the association between dose (= cumulative number of appointments or database visits) and health scales. Predictors were: COVID-19 lockdown, initial symptomatology (baseline), measurement time points, and dose. Interaction effects were also considered in the model.

## Results

### Participant flow

In total, 1,276 (n_CG_ = 523, n_IG_ = 753) patients were enrolled in PIKKO. 1,004 (n_CG_ = 424, n_IG_ = 580) participated in the initial survey by completing the baseline questionnaire. Reasons for not participating were subsequent withdrawal of commitment (n_CG_ = 33, n_IG_ = 49), death (n_CG_ = 24, n_IG_ = 8), inclusion criteria not met (n_CG_ = 1, n_IG_ = 8) or excessive health burden (n_CG_ = 16, n_IG_ = 6). CG participants who changed to the IG shortly after enrollment were also excluded from the study (*n* = 47). For some patients the reason remained unknown (n_CG_ = 15, n_IG_ = 55).

**Fig. 2:**
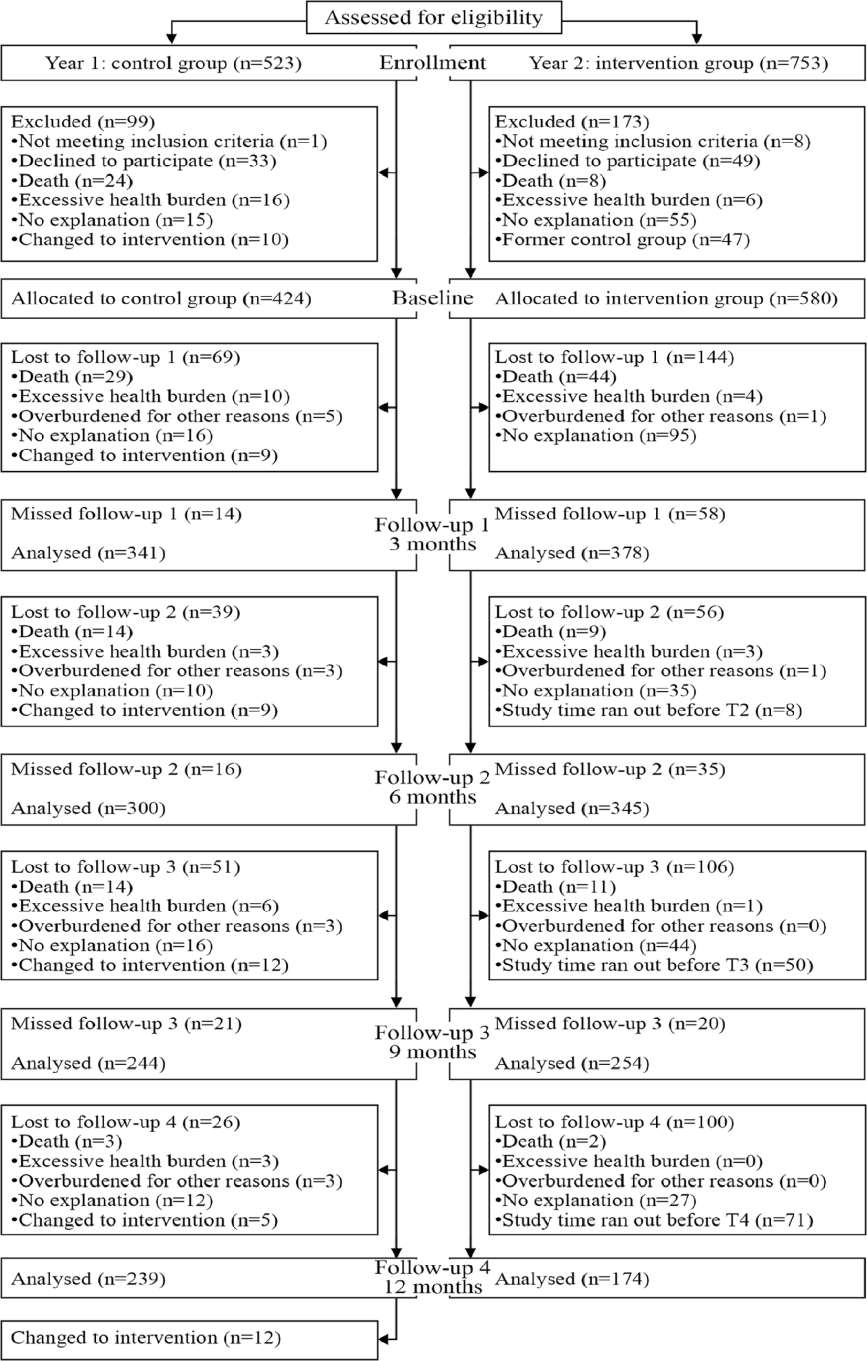
Flow chart

During the study period, further patients dropped out (for more details see Fig. 2). The dropout rates for the main outcome (follow-up 2, six months after baseline) were 25.5% (108/424) in the CG and 34.5% (200/580) in the IG. By the end of data collection, 43.6% (185/424) dropped out in CG and 70.0% (406/580; in 22.2%, 129/580, last measurement time points were after the end of the study) in IG. For the main outcome, 300 patients of the CG and 345 patients of the IG were analyzed. For all available data sets see Table 1.

### Recruitment

Recruitment of CG began on 01/11/2017 and ended on 31/10/2018, while the IG was recruited from 01/11/2018 to 31/03/2020. Data collection ended on 30/09/2020, and both recruitment periods were extended due to unexpectedly poor recruitment success, so IG with a later date could not participate in all follow-up surveys.

### Baseline data

As Table 2 shows, CG and IG have a different composition. IG patients on average were significantly (with small effect sizes) younger (IG: 58.6 ± 11.0; CG: 61.9 ± 10.7), often more had had the cancer for less than a year (CG: 68.0%, 274/403; IG: 79.1%, 424/536) and were less often on chemo-/antibody/hormone therapy at baseline (CG: 61.3%, 260/424; IG: 50.7%, 294/580). CG patients on average had significantly (with negligible effect sizes) fewer years of education (CG: 11.6 ± 2.9; IG: 12.1 ± 3.1) and were more likely to have been in school "10 years or less" (CG: 82.3%, 349/424; IG: 75.2%, 436/580), less likely to have "children living in the household" (CG: 15.8%, 66/417; IG: 23.1%, 131/566), less likely to have "cancer of the female genitalia/breast" (CG: 38.2%, 162/424; IG: 47.6%, 276/580), and more often had "leukaemia/lymphoma" (CG: 18.2%, 77/424; IG: 11.6%, 67/580) and "distant metastases" (CG: 21.5%, 91/424; IG: 14.3%, 83/580), and were less likely to report "no treatment at baseline" (CG: 11.8%, 50/424; IG: 17.6%, 102/580).

**Table 2:** Baseline socio-demographic and clinical characteristics of participants (*n* = 1,004). This table is based on the unimputed and unadjusted data

	Control group (TAU) *n* = 424	Intevention group (TAU + PIKKO) *n* = 580	Statistic
Age *m (sd)*	61.9 (10.7)	58.6 (11.0)	F(1, 1002) = 22.363, *p* < *0.001*, η^2^ = 0.022
Gender *n (%)*			
Female	257 (60.6%)	383 (66.0%)	χ^2^(1) = 3.115, p = 0.078, V = 0.056
Male	167 (39.4%)	197 (34.0%)	
School level *n (%)*			
< 10 years of school	237 (55.9%)	288 (49.7%)	χ^2^(2) = 7.624, *p* = *0.022*, V = 0.087
10 years of school	112 (26.4%)	148 (25.5%)	
> 10 years of school	75 (17.7%)	144 (24.8%)	
Education years (school + vocational) *m (sd)*	11.6 (2.9)	12.1 (3.1)	F(1, 999) = 9.290, *p* = *0.002*, η^2^ = 0.009
Marital status* n (%)*			
Single	36 (8.5%)	78 (13.5%)	χ^2^(3) = 7.478, p = 0.058, V = 0.086
Married	303 (71.6%)	389 (67.2%)	
Divorced	43 (10.2%)	67 (11.6%)	
Widowed	41 (9.7%)	45 (7.8%)	
Unknown	1 (0.2%)	1 (0.2%)	
Living with the partner* n (%)*	324 (78.8%)	430 (75.6%)	χ^2^(1) = 1.430, p = 0.232, V = 0.038
Children living in the household* n (%)*	66 (15.8%)	131 (23.1%)	χ^2^(1) = 8.023, *p* = *0.005,* V = 0.090
Financial worries* n (%)*	81 (19.5%)	91 (16.0%)	χ^2^(1) = 1.982, p = 0.159, V = 0.045
Period of the most recent illness* n (%)*			
up to 1 year (acute)	274 (68.0%)	424 (79.1%)	χ^2^(2) = 14.958, *p* = *0.001*, V = 0.126
2–5 years	94 (23.3%)	80 (14.9%)	
> 6 years	35 (8.7%)	32 (6.0%)	
Enrollment inpatient* n (%)*	280 (66.0%)	438 (75.5%)	χ^2^(1) = 10.805, *p* = *0.001*, V = 0.104
Groups of cancer* n (%)*			
Gastrointestinal (C00-25)	83 (19.6%)	131 (22.6%)	χ^2^(1) = 1.324, p = 0.250, V = 0.036
Lung and larynx (C32-34)	67 (15.8%)	71 (12.2%)	χ^2^(1) = 2.619, p = 0.106, V = 0.051
Female genitals / breast (C50-56)	162 (38.2%)	276 (47.6%)	χ^2^(1) = 8.760, *p* = *0.003*, V = 0.093
Male genitals (C61-62)	34 (8.0%)	31 (5.3%)	χ^2^(1) = 2.893, p = 0.089, V = 0.054
Leukaemia, lymphoma (C81-96)	77 (18.2%)	67 (11.6%)	χ^2^(1) = 8.707, *p* = *0.003*, V = 0.093
Other	74 (17.5%)	58 (10.0%)	χ^2^(1) = 11.915, *p* = *0.001*, V = 0.109
Cancer status* n (%)*			
Tumour spread	187 (44.1%)	282 (48.6%)	χ^2^(1) = 2.008, p = 0.157, V = 0.045
Lymph node metastases	97 (22.9%)	122 (21.0%)	χ^2^(1) = 0.488, p = 0.485, V = 0.022
Distant metastases	91 (21.5%)	83 (14.3%)	χ^2^(1) = 8.745, *p* = *0.003*, V = 0.093
Relapse	17 (4.0%)	13 (2.2%)	χ^2^(1) = 2.641, p = 0.104, V = 0.051
Cancer treatment* n (%)*			
CT, ABT, HT	260 (61.3%)	294 (50.7%)	χ^2^(1) = 11.193, *p* = *0.001*, V = 0.106
Radiotherapy	66 (15.6%)	82 (14.1%)	χ^2^(1) = 0.397, p = 0.528, V = 0.020
Surgery, past and present	167 (39.4%)	200 (34.5%)	χ^2^(1) = 2.540, p = 0.111, V = 0.050
Artificial nutrition	6 (1.4%)	10 (1.7%)	χ^2^(1) = 0.149, p = 0.699, V = 0.012
Rehabilitation	58 (13.7%)	75 (12.9%)	χ^2^(1) = 0.119, p = 0.730, V = 0.011
No treatment	50 (11.8%)	102 (17.6%)	χ^2^(1) = 6.400, *p* = *0.011*, V = 0.080

*CT* chemotherapy, *ABT* Antibody therapy, *HT* Hormone therapy

### Numbers analyzed

As shown in Fig. 2, the number of participants included in the analyses decreased over the measurement time points. At follow-up 1 (three months) *n* = 341 participants could be analyzed in the CG and *n* = 378 in the IG. At follow-up 2, the main outcome (six months), *n* = 300 participants could be analyzed in the CG and *n* = 345 in the IG (338 per group were targeted). At follow-up 3 (nine months) *n* = 244 participants could be analyzed in the CG and *n* = 254 in the IG. At follow-up 4 (one year) *n* = 239 participants could be analyzed in the CG and *n* = 174 in the IG.

Only IG data were used to analyze the utilization of the intervention modules (exception: ring folder). The maximum number of potential PN users was 436 patients (all with baseline data who participated in at least one follow-up), SCS service users was 580 (all baseline data, including those who no longer participated in follow-ups) and knowledge database users was 627 (entire IG with baseline data plus switchers from CG). Dose effects of SCS counseling could be determined from 72 patients (SCS documented users).

### Outcomes

### Main Analyses

  Analyses of imputed and regression-adjusted data (Table 3) showed no advantage of IG over CG in any of the questionnaires used. The measured differences (Table 3) in mental health-related quality of life at six (CG: 46.38 ± 8.01; IG: 44.92 ± 6.45) and nine (CG: 46.90 ± 7.05; IG: 45.43 ± 5.49) months and in depression at three (CG: 7.67 ± 3.15; IG: 8.31 ± 2.61), six (CG: 7.24 ± 3.18; IG: 8.08 ± 2.58), and nine (CG: 7.16 ± 2.80; IG: 7.43 ± 2.85) months even seem to favor the CG.

**Table 3:** Comparative statistics (mean (sd), 95% confidence interval), Hedges' g of the comparison (IG and CG) of imputed and regression-adjusted data and percent of missing before imputation

	IG	CG	Hedges’ g	Missing
Mental HRQoL (SF12)^1^				
Baseline	42.34 (10.27), 41.50–43.17	42.18 (9.85), 41.24–43.12	0.015	5.9%
Follow-up 1 (3 months)	43.89 (6.51), 43.23–44.54	44.80 (7.91), 43.97–45.64	-0.141	31.8%
Follow-up 2 (6 months)	44.92 (6.45), 44.24–45.60	46.38 (8.01), 45.47–47.29	-0.226*	38.2%
Follow-up 3 (9 months)	45.43 (5.49), 44.75–46.10	46.90 (7.05), 46.02–47.79	-0.269**	52.8%
Follow-up 4 (12 months)	45.41 (7.28), 44.33–46.49	46.38 (8.79), 45.27–47.49	-0.132	60.4%
Physical HRQoL (SF12)^1^				
Baseline	38.46 (10.00), 37.65–39.28	38.50 (9.65), 37.58–39.42	-0.004	5.9%
Follow-up 1 (3 months)	38.32 (6.73), 37.65–39.00	39.29 (7.93), 38.45–40.13	-0.143	31.8%
Follow-up 2 (6 months)	38.89 (6.61), 38.19–39.58	40.02 (7.89), 39.13–40.91	-0.171	38.2%
Follow-up 3 (9 months)	40.15 (5.66), 39.45–40.85	40.70 (6.99), 39.82–41.57	-0.096	52.8%
Follow-up 4 (12 months)	42.11 (6.85), 41.10–43.13	41.32 (8.34), 40.26–42.37	0.117	60.4%
Depression (PHQ-9)^2^				
Baseline	8.48 (4.06), 8.15–8.81	8.47 (3.89), 8.10–8.84	0.003	5.0%
Follow-up 1 (3 months)	8.31 (2.61), 8.05–8.57	7.67 (3.15), 7.34–8.01	0.243**	32.2%
Follow-up 2 (6 months)	8.08 (2.58), 7.80–8.35	7.24 (3.18), 6.88–7.60	0.326***	37,9%
Follow-up 3 (9 months)	7.78 (2.20), 7.51–8.05	7.16 (2.80), 6.80–7.51	0.285**	52.0%
Follow-up 4 (12 months)	7.43 (2.85), 7.01–7.86	7.43 (3.45), 6.70–7.87	0	60.0%
Anxiety (GAD-7)^3^				
Baseline	6.09 (3.45), 5.81–6.37	6.03 (3.32), 5.72–6.35	0.017	4.2%
Follow-up 1 (3 months)	5.70 (2.23), 5.47–5.92	5.62 (2.69), 5.33–5.90	0.037	32.5%
Follow-up 2 (6 months)	5.46 (2.20), 5.23–5.70	5.39 (2.72), 5.08–5.70	0.034	37.8%
Follow-up 3 (9 months)	5.38 (1.88), 5.15–5.61	5.35 (2.40), 5.05–5.65	0.018	52.1%
Follow-up 4 (12 months)	5.45 (2.42), 5.09–5.81	5.49 (2.94), 5.12–5.87	-0.017	59.5%
Self-efficacy (GSE)^4^				
Baseline	28.51 (5.38), 28.07–28.94	28.63 (5.18), 28.14–29.12	-0.023	5.8%
Follow-up 1 (3 months)	28.88 (3.70), 28.51–29.26	29.00 (4.30), 28.54–29.45	-0.030	31.8%
Follow-up 2 (6 months)	29.14 (3.62), 28.76–29.53	29.27 (4.25), 28.79–29.76	-0.036	38.4%
Follow-up 3 (9 months)	29.28 (3.10), 28.90–29.66	29.47 (3.77), 29.00–29.94	-0.061	52.3%
Follow-up 4 (12 months)	29.30 (3.62), 28.76–29.84	29.58 (4.43), 29.02–30.14	-0.077	59.7%
Health literacy (HLS-EU-Q47)^5^				
Baseline	33.08 (6.25), 32.57–33.59	32.99 (6.03), 32.42–33.56	0.014	25.2%
Follow-up 1 (3 months)	33.92 (4.21), 33.49–34.34	34.06 (4.97), 33.53–34.58	-0.033	45.0%
Follow-up 2 (6 months)	34.56 (4.13), 34.12–34.99	34.80 (4.95), 34.24–35.36	-0.058	48.9%
Follow-up 3 (9 months)	35.00 (3.54), 34.57–35.44	35.22 (4.38), 34.67–35.77	-0.061	59.2%
Follow-up 4 (12 months)	35.25 (4.29), 34.61–35.89	35.32 (5.23), 34.65–35.98	-0.016	65.7%

^1^ SF-12 ranged from 0 to 100, high score indicates high HRQoL, ^2^ PHQ-9 ranged from 0 to 27, high score indicates high depression (< 5: no depression), ^3^ GAD-7 ranged from 0 to 21, high score indicates high anxiety (< 5: no anxiety), ^4^ GSE ranged from 10 to 40, high score indicates high self-efficacy, ^5^ HLS-EU-Q47 ranged from 0 to 50, high score indicates high health literacy, *** *p* < 0.001, ** *p* < 0.01, *p < 0.05

### Utilization and evaluation of the intervention modules by the participants

The intervention modules were used to a high degree by the patients (Table 4).

**Table 4:** Data on the IG's utilization and evaluation of the intervention modules (the folder was also used by the CG)

	Folder	Patient navigator	Offers of the Saarland Cancer Society		Knowledge database
			Counseling^1^	Courses^2^	
Missing data [%]	T1: 28.4% T2: 35.8% T3: 50.4% T4: 58.9%	T1: 28.4% T2: 35.8% T3: 50.4% T4: 58.9%	T1: 62.0% T2: 65.1% T3: 74.3% T4: 82.4%		T1: 29.7% T2: 36.5% T3: 50.4% T4: 59.1%
Total users [n (%)]	IG: 368/436^3^ (84.4%) CG: 269/355^3^ (75.8%)	382/436^3^ (87.6%)	172/580 (29.7%)	127/580 (21.9%)	413/627^4^ (65.9%)
Usage frequency [m (sd), max]	N.A	5.07 (5.73), 41	2.88 (2.63), 15	1.61 (2.30), 12	2.28 (2.61), 30
Overall rating^5^ [m (sd)]	N.A	1.96 (0.81)	1.39 (0.77)	NW: 1.07 (1.90) AC: 1.17 (0.32) Y: 1.29 (0.69) QG: 1.35 (0.69) MT: 1.64 (0.76) NC: 1.65 (1.08) NA: 1.97 (1.01)^6^	2.16 (0.74)
Which module did you use most often?^7^	IG: 244/436 (56.0%)	175/436 (40.1%)	139/436 (31.9%)		156/436 (35.8%)
Which module did you find most helpful?^7^	IG: 177/436 (40.6%)	190/436 (43.6%)	137/436 (31.4%)		146/436 (33.5%)

^1^psychological and social-legal ^2^including nutrition counseling and lectures; ^3^number of participants with at least one follow-up; ^4^number of participants with a theoretical access to the database (activation during their initial meeting with the PN); ^5^rating according to school grade (1 = very good to 6 = insufficient); ^6^range, as each course (AC = art and creative course, MT = music therapy, NA = nutrition advice via telephone, NC = nutrition course, NW = Nordic walking, QG = QiGong, Y = yoga) was rated individually ^7^All patients who answered the question in the affirmative at least one follow-up visit were counted (double answers were possible)

The folder was used by both CG (75.8%, 269/355) and IG (84.4%, 368/436), in most cases to file medical and hospital discharge letters (IG: 62.6%, 273/436; CG: 61.1%, 217/355). The folder was rated as supportive by 71.1% of IGs (295/415) and 70.0% of CGs (226/323).

The PN was contacted by telephone or directly after the initial meeting by 87.6% (382/436) of the IG with follow-up investigation. On average, the PN was consulted five times (5.07 ± 5.69). The most frequent topics of consultation were socio-legal issues (58.0%, 253/436), additional offers e.g. of the SCS (56.2%, 245/436) and topics concerning cancer therapy (48.9%, 213/436). PN was used by 39.0% (*N* = 170/436) of patients for psychological support. The overall rating of the PN across all survey time points and all participants was “good” (1.96 ± 0.81). In particular, the exclusive time for the patient (2.29 ± 0.76), understanding (2.37 ± 0.70) and compassion (2.33 ± 0.70) of PN were perceived as better than in normal consultations with physicians (rating: 1 = much better, 2 = better, 3 = equal, 4 = poor, 5 = much worse).

At least one of the SCS offers was used by 39.8% of the IG (231/580), 29.7% (172/580) used psycho-social counseling, and 21.9% (127/580) took one or more of the course units. The course units were used (related to the survey period) with varying frequency, ranging from one-time participation (nutrition course 1.04 ± 0.21) to an average of three times per participant (art and creative course 3.13 ± 2.70). The psycho-social counseling was used by many, but the frequency of use is known of only a few (*N* = 72). Of those, only 6.9% (5/72) had seven or more appointments during the year of the PIKKO survey, 31.9% (23/72) had 3–6 appointments, 25.0% (18/72) had two appointments and 36.1% (26/72) had one appointment. The overall offer was rated by the patients with “good” (1.99 ± 0.83) and the consultation even achieved “very good” (1.39 ± 0.77).

Of all patients who had an initial meeting with the PN and could have obtained activation for the knowledge database at that time, 65.9% (413/627) visited it. On average, patients used the knowledge database twice (2.16 ± 2.61), although there were some very frequent uses (up to 30 times). Aspects such as therapy and treatment (41.6%, 172/413), nutrition (40.9%, 169/413), side effects (34.1%, 141/413), physical activity (32.0%, 132/413) cancer and cancer development (31.5%, 130/413), and insurance (30.0%, 124/413) were most frequently searched. Only 18.4% (76/413) of users searched for information on psychological support. Database users who also participated in follow-up surveys rated the database as “good” (2.16 ± 0.74).

The majority of patients (56.0%, 244/436) reported using the folder most often, of all the aspects of the PIKKO intervention. Conversely, the PN was rated “most helpful” by most patients (43.6%, 190/436).

### Analyses of dose effects

No significant associations were found regarding cumulative frequency of visits to the PNs.

**Fig. 3:**
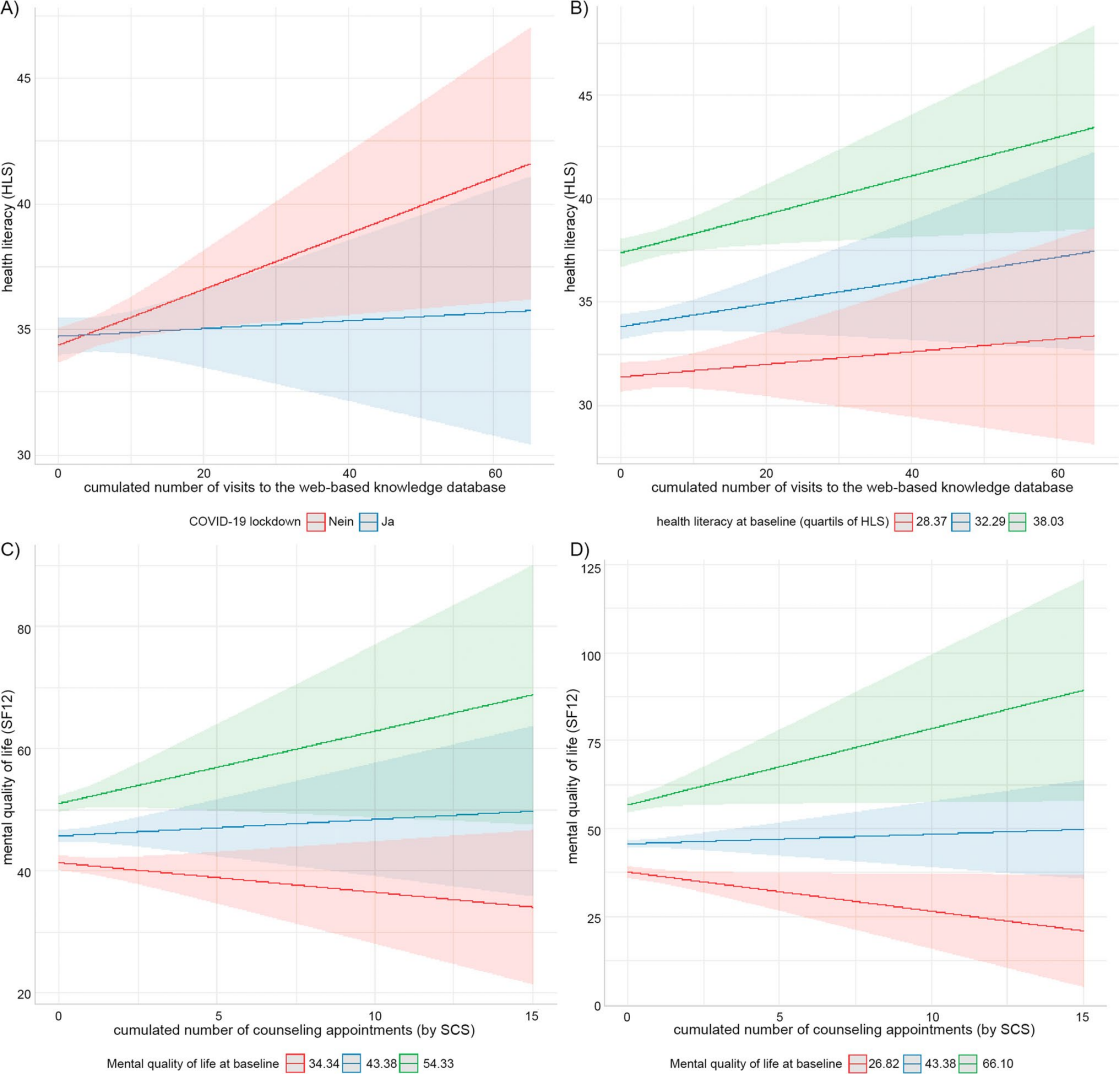
Predicted values of (**a**, **b**) health literacy, HLS, or (**c**, **d**) mental health-related quality of life, SF12, as a function of cumulated number of (**a**, **b**) visits to the web-based knowledge database or (**c**, **d**) counseling appointments by SCS with (**a**) COVID-19 lockdown, (**b**) baseline health literacy, quartils, (**c**) mental health-related quality of life or (**d**) mental health-related quality of life (with estimated Johnson-Neyman interval) as a predictor

The analysis of user data of the knowledge database revealed two significant interaction effects. First, under non-COVID-19 lockdown conditions, an increase in health literacy could be expected with more frequent use (Fig. 3A). Second, the increase in health literacy was strongest for patients with initially high health literacy (Fig. 3B).

Dose data of counseling by SCS was available for 72 patients (only five had seven or more appointments). The cumulative number of counseling appointments offered by the SCS improved in mental health-related quality of life for those with high baseline scores of this health metric (Fig. 3C and 2D).

### Harms

No patient reported any harm caused by using the intervention modules or the evaluation.

## Discussion

The aim of PIKKO was to support cancer patients with additional services to improve their health-related quality of life and health literacy and to reduce negative side effects such as depression and anxiety. In order to investigate these effects, various measurements of IG and CG were taken and compared at different time points using regression-adjusted analyses of differences.

The expected improvements of the intervention modules on health-related quality of life (HRQoL), depressiveness, anxiety, self-efficacy, and health literacy were not observed in the overall sample. However, module use and patient satisfaction were high. Regarding mental HRQoL and depression, the CG had an advantage that disappeared after twelve months. The results of the PIKKO questionnaire were inconsistent with the subjective positive ratings of the individual modules by the patients. Comparable results were obtained from patients who attended an outpatient cancer counseling center in Saxony, Germany [41]. Here, too, no significant differences in measures of depression, anxiety, and HRQoL were found over a 4-month period, although the counseling services were rated positively. On the other hand, Lingens et al. showed that depressive symptoms, HRQoL, and well-being were improved in cancer patients by brief psycho-social support [42]. But, our results should not be misinterpreted as ineffectiveness of the intervention. Rather, the results are likely due to the various limitations of the study, in particular the aggravating conditions caused by the COVID-19 lockdown, the lack of randomization, and the insufficient dosage of voluntary utilization, especially of psycho-social counseling. The distinction between use of psychological and socio-legal counseling could not be differentiated for methodological reasons.

The analyses of dose effects showed a tendency towards increasing improvements in patients with initially higher health literacy levels through greater use of the knowledge database. Higher initial health literacy enables better reception and interpretation of educational elements / patient information via the associated information processing skills. Therefore, we interpret that the patient group with higher initial competence experienced greater benefits due to more effective and efficient processing of the new information from the knowledge database.

Furthermore, these analyses showed a tendency towards increasing improvements in patients with initially higher mental HRQoL through more frequent use of SCS counseling. Patients with initially low HRQoL were more likely to be in ongoing acute therapy (e.g., chemotherapy), which may have limited their willingness or access to additional psycho-social services. Patients not currently in ongoing oncological therapy could therefore benefit more from SCS services. For optimal use of such services, we recommend an initial needs screening as a systematic management tool.

### Limitations

For ethical and service reasons, randomization in the conventional sense could not be performed. Instead, the two groups were part of the study consecutively (see further explanations in the methods section under "Randomization").

The PIKKO sample had higher baseline scores for depression and anxiety than that of comparable studies [43]. It is possible that patients with increased anxiety and depression were more motivated to participate in the PIKKO study.

Due to the sequential recruitment of the groups as well as the difficulties in recruitment, a selection bias can be assumed. In addition, the IG was recruited at the inpatient setting and consisted of more individuals with a recently diagnosed or recurrent cancer.

With its relatively mild inclusion criteria, PIKKO aimed to represent a naturalistic picture of cancer health care. However, different cancer types, stages, and severities, and socio-demographic characteristics [11, 44] may have very different needs (especially regarding psycho-social counseling) at different points in time. These different needs were not screened nor documented. Ross et al. also failed to show any effects of a complex intervention, pointing to a lack of screening and a resulting overly wide variation in circumstances as one reason [45]. Thus, it remains an open question exactly how many patients developed counseling needs and when, also whether all who were in need received counseling. It is possible that the study's measurement time points did not correspond with actual use of psycho-social counseling. Ernst et al. showed that cancer patients in need of advice sought outpatient help at a cancer-counseling center an average of 21 months after diagnosis [46]. About 30% were then in a disease-free or treatment-free period, which is also indicated by Rösler et al. and in internal, unpublished data of the SCS [46, 47]. Transferred to PIKKO, the support services (especially SCS counseling) would become attractive to many of the patients later, when they have completed their inpatient treatment a long time after the last survey appointment.

Another unexpected limitation was the help-seeking behavior of the CG. Unfortunately, it was not documented whether participants of the CG found support outside PIKKO.

The measurement tools used, specifically the characteristics they targeted, could also be considered a limitation. PN may improve informed decision making rather than HRQoL or depressiveness. In general, measurement tools based on self-report by cancer patients undergoing treatment and covering a past period of three months may be inaccurate or biased.

Lastly, the dosage of intervention modules use may have been a limitation, as utilization was voluntary. In particular, psychotherapeutic interventions designed to improve depression and anxiety and mental HRQoL have been shown to require seven or more sessions to show results on HRQoL, depression, or anxiety scores [48–50].

### Generalizability and learnings

Although the approach was to distribute PIKKO as broadly as possible and to test it in the real healthcare landscape, this very endeavor proved to be a limitation in establishing clear effects. Also, the sample may not have represented the true cancer landscape. For example, many more patients were included who had cancer of female genitals / breast or lung / larynx than the German cancer incidence rates would suggest. Although this limits the generalizability of the results, patients gratefully and willingly use the PIKKO interventions offered. Significant influences on the examined outcomes were not demonstrated. However, there were tendencies towards significance at higher doses of psycho-social counseling, which should be investigated in follow-up studies. Improvement in health literacy with increased use of the database was observed. For this reason, this educational eHealth element remains available beyond the end of the project and can be adapted to specific settings or research projects. The integration of such an information tool as a supporting eHealth element in oncology patient care is recommended. Furthermore, a variety of structural knowledge was gained in the course of PIKKO, which is highly relevant for the planned nationwide introduction of "Patient Navigators" by German health policy [51, 52].

## Data Availability

All data are available on request from the corresponding author.
